# A systematic review and meta-analysis of randomized controlled trials comparing low-dose versus standard-dose computed tomography-guided lung biopsy

**DOI:** 10.1186/s13019-024-02792-x

**Published:** 2024-05-22

**Authors:** Teng Li, Guanghui Xu, Wenjun Li, Yun Liu

**Affiliations:** 1 Department of Interventional Radiology, The People’s Hospital of Weifang, 151 Guangwen Street, Weifang, Shandong 261041 China; 2 Department of Hematology, The People’s Hospital of Weifang, 151 Guangwen Street, Weifang, Shandong 261041 China

**Keywords:** Low-dose computed tomography, Lung biopsy, Diagnostic accuracy

## Abstract

**Background:**

Despite the existence of several Randomized Controlled Trials (RCTs) investigating Low-Dose Computed Tomography (LDCT) as a guide in lung biopsies, conclusive findings remain elusive. To address this contention, we conducted a systematic review and meta-analysis to evaluate the efficacy and safety of LDCT-guided lung biopsies.

**Methods:**

A comprehensive search across major databases identified RCTs comparing the effectiveness of LDCT-guided with Standard-Dose Computed Tomography (SDCT)-guided lung biopsies. Subsequently, we utilized a random-effects model meta-analysis to assess diagnostic accuracy, radiation dose, operation duration, and clinical complications associated with these procedures.

**Results:**

Out of 292 scrutinized studies, six RCTs representing 922 patients were included in the final analysis. Results indicated the differences between the LDCT and SDCT groups were not different with statistical significance in terms of diagnostic accuracy rates (Intent-to-Treat (ITT) populations: Relative Risk (RR) 1.01, 95% Confidence interval [CI] 0.97–1.06, *p* = 0.61; Per-Protocol (PP) populations: RR 1.01, 95% CI 0.98–1.04, *p* = 0.46), incidence of pneumothorax (RR 1.00, 95% CI 0.75–1.35, *p* = 0.98), incidence of hemoptysis (RR 0.95, 95% CI 0.63–1.43, *p* = 0.80), and operation duration (minutes) (Mean Differences [MD] -0.34, 95% CI -1.67-0.99, *p* = 0.61). Notably, LDCT group demonstrated a lower radiation dose (mGy·cm) with statistical significance (MD -188.62, 95% CI -273.90 to -103.34, *p* < 0.0001).

**Conclusions:**

The use of LDCT in lung biopsy procedures demonstrated equivalent efficacy and safety to standard methods while notably reducing patient radiation exposure.

**Supplementary Information:**

The online version contains supplementary material available at 10.1186/s13019-024-02792-x.

## Introduction

Computed Tomography (CT)-guided lung biopsies represent a well-established and commonly utilized technique essential for diagnosing lung lesions. This method has demonstrated diagnostic accuracy within the range of 88–97% [1–3], and a major complication rate of approximately 5.7% [4, 5]. The increasing prevalence of lung cancer, improved detection rates for asymptomatic lung nodules, and the growing demand for tissue sampling for advanced molecular profiling and genomic analysis have collectively fueled the need for CT-guided lung biopsy procedures [6].However, implementing CT-guided biopsies requires repeated CT scans to locate lesions, refine needle placement, and assess potential complications. Consequently, this procedure exposes patients to elevated radiation doses, raising concerns regarding potential long-term risks, particularly among those undergoing repeated scans or those with heightened susceptibility to radiation-related complications [7, 8].

To address this concern, the adoption of a Low-Dose CT (LDCT) protocol has emerged as a viable solution. LDCT utilizes specialized scanning protocols that significantly reduce the radiation dose administered to patients without compromising essential image quality. This reduction in radiation exposure helps mitigate potential health risks associated with radiation exposure. LDCT is routinely used for lung cancer screenings, especially among high-risk individuals [9, 10]. Furthermore, various studies have confirmed its suitability for guiding lung biopsy procedures.

Several Randomized Controlled Trials (RCTs) have been conducted to evaluate the effectiveness of a LDCT protocol compared to a Standard-Dose CT (SDCT) protocol for guiding lung biopsy procedures [11–17]. The findings of these trials have been promising. However, it should be noted that all these trials were single-institutional with limited sample sizes, and some displayed inadequate methodological rigor. In a previous meta-analysis by Zhang et al. [18], it was determined that LDCT-guided biopsy could achieve comparable diagnostic efficacy with reduced radiation doses. Yet, this meta-analysis only included three RCTs and three non-RCTs, with one RCT [11] excluded from the pooled data. Additionally, two recent RCTs [16, 17] have been published, which may offer new perspectives on this topic. To assess the effectiveness and safety of the LDCT protocol, a systematic review and meta-analysis of RCTs was conducted.

## Materials and methods

This meta-analysis was conducted in strict adherence to the guidelines outlined by the Preferred Reporting Items for Systematic Reviews and Meta-Analyses (PRISMA) statement [19] and was promptly registered on PROSPERO (www.crd.york.ac.uk/prospero/) with registration number CRD42023454261. Since all analyses were derived from previously published studies, ethical approval as well as patient consent were deemed unnecessary.

### Literature search and study selection

Two authors, Teng Li and Yun Liu, independently executed a comprehensive search across multiple databases, including PubMed, Embase, Cochrane Library, Web of Science, and ClinicalTrials.gov, to identify relevant articles. No language restrictions were applied, and the search encompassed studies published until August 20, 2023. The primary terms used in our search strategy were “low-dose computed tomography,” “lung,” and “biopsy,” combined with “randomized controlled trial.” Additionally, we scrutinized the reference lists of identified articles to uncover any additional qualifying studies for inclusion in our analysis.

The inclusion criteria for this study were twofold: [1] RCTs comparing LDCT-guided and SDCT-guided lung biopsies (SDCT protocols encompassed conventional lung scanning schemes which were applied on various CT scanners. LDCT protocols involved the modification of parameters, such as the tube current and voltage, to levels below those of standard protocols, augmenting the helical pitch or implementing tin filtration), and [2] studies presenting at least one extractable outcome. Conversely, the exclusion criteria included: [1] animal studies [2], studies involving cone beam CT or CT fluoroscopy [3], studies where variables (e.g., utilization of a navigation system or iterative algorithms) other than scanning parameters differed between groups, and [4] studies providing inadequately detailed data.

Two authors, Guanghui Xu and Wenjun Li, meticulously scrutinized titles and abstracts obtained from the database searches. Should either author find a title and abstract meeting the inclusion criteria, the full text of the study was obtained. The inclusion of an article required consensus between both authors, with disagreements resolved through discussion or, if necessary, consultation with a third author, Yun Liu.

### Data extraction and quality assessment

Data were extracted by two independent authors, Guanghui Xu and Wenjun Li, utilizing a comprehensive data extraction sheet encompassing various parameters. These parameters included fundamental characteristics of the studies such as authors, year of publication, country of study, and diameter of needles used. Moreover, patient-related characteristics like sample size, mean age, lesion size, and Body Mass Index (BMI) were meticulously recorded. Procedure-related characteristics including scanning protocols, diagnostic accuracy rates, radiation dose, operative duration, and complications were meticulously documented. The potential bias of the RCTs was assessed using the Cochrane risk of bias tool [20]. In this analysis, trials with a low risk of bias in five or more items were deemed to have an overall low risk of bias. Any discrepancies were resolved through either discussion or consultation with a third reviewer, Yun Liu.

### Endpoints and definitions

The primary aim of the current analysis involved evaluating diagnostic accuracy, assessed through Intent-to-Treat (ITT) and Per-Protocol (PP) analyses. Secondary endpoints included radiation dose, operation duration, and clinical complications. Diagnostic accuracy, as denoted by the ITT analysis, constituted the aggregate of true positives and true negatives for all cases included in the study. Similarly, the diagnostic accuracy, evaluated by PP analysis, took into account the total of true positives and true negatives for all instances with a conclusive diagnosis. The dose of radiation was measured using the Dose-Length Product (DLP) in mGy·cm. The operation duration was quantified as the period in minutes from the administration of local anesthesia up to the completion of CT scan post-biopsy. Clinical complications included pneumothorax and hemoptysis.

### Statistical analysis

For dichotomous outcomes (diagnostic accuracy and clinical complications), the Relative Risk (RR) with 95% Confidence Intervals (CI) was computed. For continuous outcomes (radiation dose and operation duration), Mean Differences (MD) with 95% CI were determined. Median values (interquartile range or range) were transformed into mean values (standard deviation). Given the variation in LDCT and SDCT protocols across different studies, consideration for potential discrepancies between studies due to the variable methodology was essential. As a result, a random-effects model was considered most appropriate. The presence of heterogeneity was assessed using the I^2^ statistic, with I^2^ > 50% signifying significant heterogeneity [21]. In cases where significant heterogeneity was present, sensitivity analyses were implemented to determine the influence of individual studies on the overall result. This was done by consecutively omitting one study at a time. And concurrently conducting subgroup analyses to detect sources of heterogeneity. Due to the limited number of studies (less than ten), an evaluation of publication bias was not conducted [22]. A P-value less than 0.05 indicated statistically significant. Statistical analyses were performed utilizing Review Manager version 5.3 (The Cochrane Collaboration, Software Update, Oxford, UK).

## Results

### Literature search, study characteristics and quality assessment

**Fig. 1:**
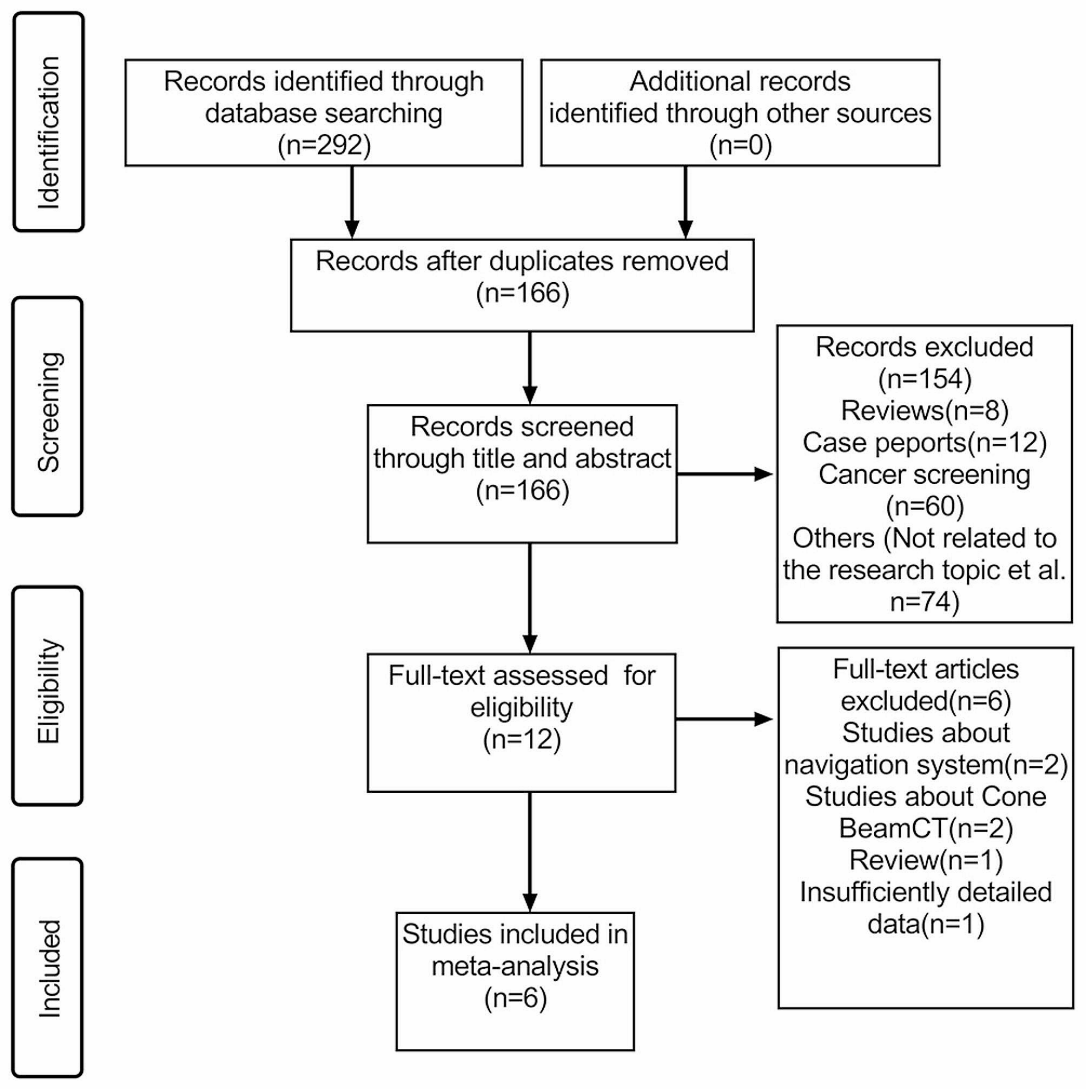
Flowchart of the search and selection process

A comprehensive search across multiple databases initially yielded 292 articles: 94 from PubMed, 73 from Embase, 77 from Cochrane Library, 44 from Web of Science, and 4 from ClinicalTrials.gov. After removing duplicates, the count of unique articles decreased to 166. Subsequently, 154 articles, including reviews, letters, animal studies, or those containing content irrelevant to this specific research were excluded. Following the application of inclusion and exclusion criteria, an additional six full-text articles were discarded. Consequently, six articles met the criteria of eligibility and were then included in this meta-analysis [11, 12, 14–17]. A detailed flowchart outlining the search and selection process is presented in Fig. 1.

The selected studies were published between 2013 and 2023. All trials were single-center, with five conducted in China [12, 14–17] and one in Italy [11]. The sample size ranged from 52 to 271 patients, encompassing 498 patients who underwent LDCT-guided lung biopsies and 424 patients who underwent SDCT-guided lung biopsies. In an effort to reduce radiation dose, four studies opted to lower the tube current [11, 12, 15, 17], while the remaining two implemented tin filtration without reducing the tube voltage and current [14, 16]. Detailed characteristics of the studies included in this meta-analysis are presented in Table 1.

**Table 1:** Characteristics of included studies

NO	**Author**	Year	Country	Characteristics of lesions	LDCT group							SDCT group						
					**Patients** **(male)**	Age (years)	Lesion size (mm)	BMI (kg/m^2^)	Lesion depth (mm)	Tube voltage (kV)	Tube current (mAs)	Patients (male)	Age (years)	Lesion size (mm)	BMI (kg/m^2^)	Lesion depth (mm)	Tube voltage (kV)	Tube current (mA)
1	Grasso	2013	Italy	Solid or ground glass opacitie	27 (16)	69.8 ± 10.7	34.4 ± 20.1	-	48.2%>20mm	100	35	25 (15)	66.2 ± 12.2	33.1 ± 20.1	-	40%>20mm	100	100
2	Meng	2013	China	Solid component > 50%	46 (27)	63.7 ± 12.9	32.5 ± 15.7	19.8 ± 6.4	32.00 + 11.03	120	10	44 (24)	62.7 ± 12.9	31.2 ± 15.7	20.3 ± 5.4	31.50 + 12.00	120	200
3	Li	2019	China	Size < 3cm	140 (97)	61 ± 15	22 ± 9	27.1 ± 5.4	23 ± 20	100kV with tin filtration	70	70 (44)	66 ± 17	19 ± 8	26.4 ± 4.1	22 ± 17	110	50
4	Fu	2020	China	Solid component > 80% size ≥ 5mm	135 (92)	62.6 ± 11.1	44.6 ± 26.6	23.1 ± 3.7	12.6 ± 14.3	120	15	136 (89)	61.2 ± 12.4	44.1 ± 24.5	22.8 ± 3.1	17.9 ± 16.9	120	150
5	Zhang	2022	China	Not mentioned	50 (29)	60.6 ± 9.7	44.4 ± 30.6	23.0 ± 4.4	14.50 ± 14.81	130kV with tin filtration	56	49 (31)	63.9 ± 8.8	37.6 ± 17.7	23.5 ± 3.1	18.64 ± 14.96	130	56
6	Li	2023	China	Solid, size > 8 mm	100 (68)	63.7 ± 10.5	24.8 ± 4.2	23.1 ± 3.6	15%≥30 mm	120	15	100 (62)	61.1 ± 13.0	23.5 ± 5.0	22.8 ± 3.2	25%≥30 mm	120	150

BMI: Body Mass Index; Lesion depth: Lesion-to-pleural surface distance

**Fig. 2:**
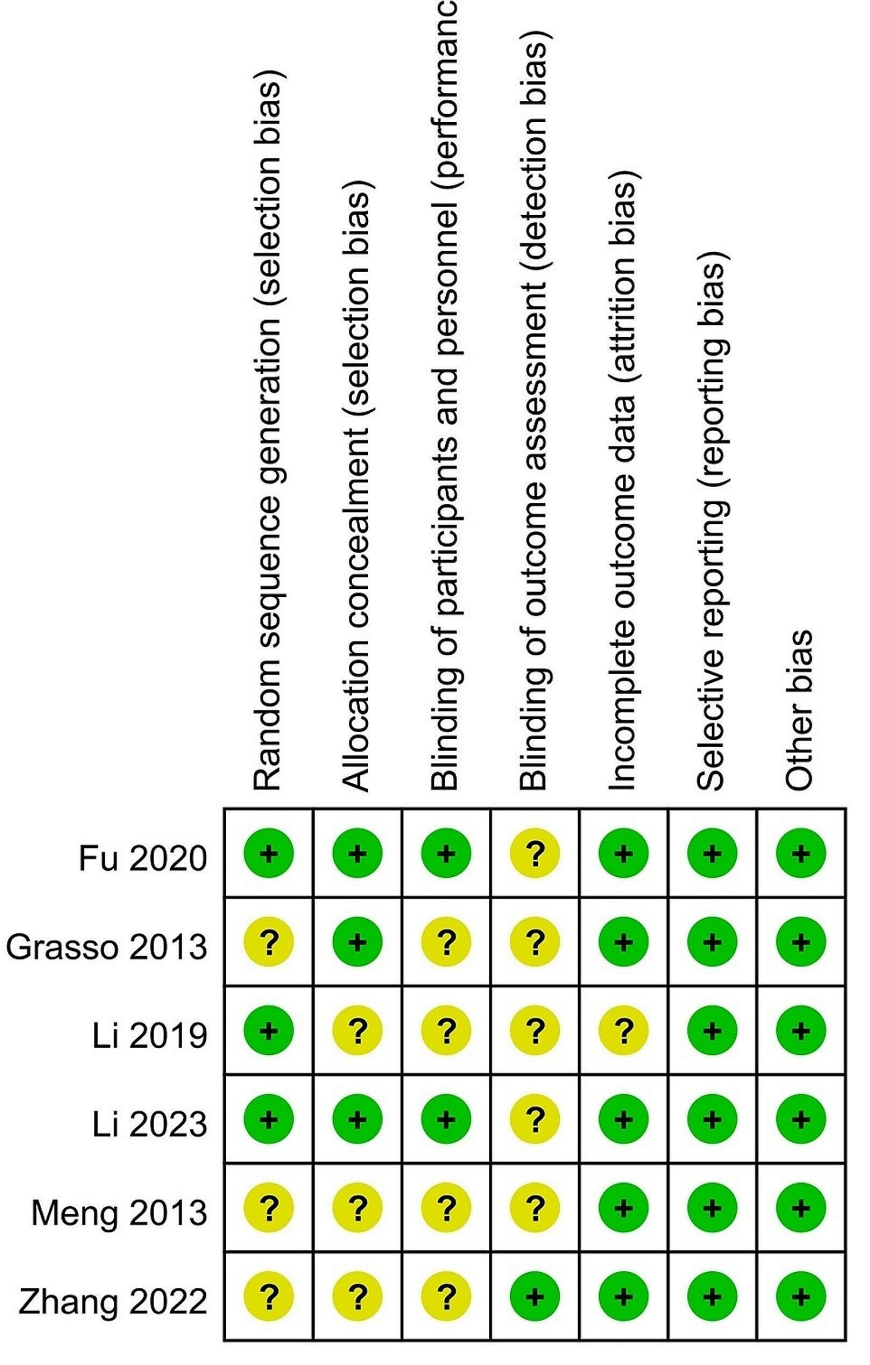
Summary of the risk of bias

**Fig. 3:**
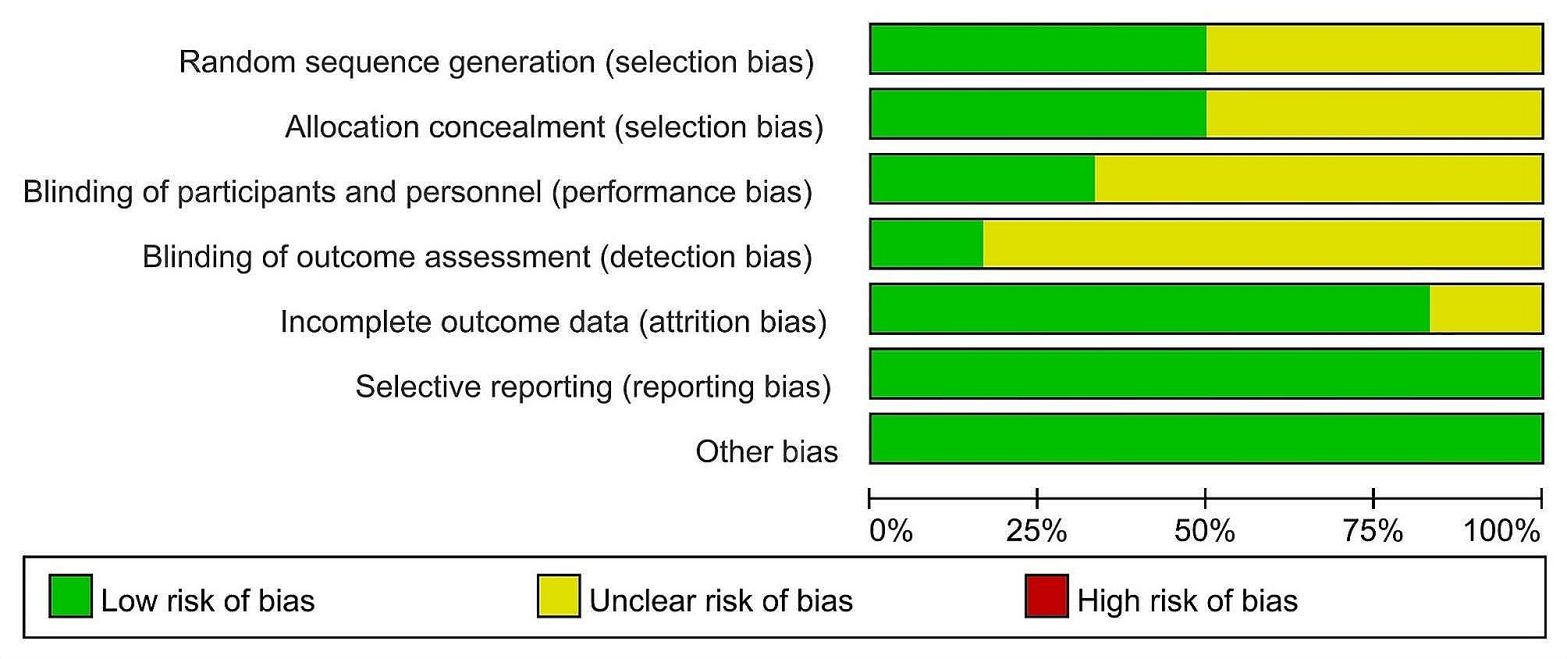
Graphical representation of the risk of bias

Each selected study employed a traditional parallel group design. All studies utilized an 18-gauge needle to conduct core biopsy with a coaxial technique. Additionally, one study [11] utilized an optical navigation system, while the other studies did not employ any navigation systems. Procedures across all trials were performed by skilled interventional radiologists, ensuring optimal results without potential learning curve effects during the trials. Among the six selected studies, three lacked clarity on sequence generation details [11, 12, 16]. The remaining studies utilized appropriate randomized sequence generation techniques [14, 15, 17]. Allocation concealment was achieved through the use of appropriately sealed envelopes in three studies [11, 15, 17]. Only two trials involved patient blinding [15, 17] and one enlisted blinding of data analysts [16]. Two trials exhibiting low risk of bias in no less than 5 items were categorized as high-quality studies [15, 17]. Summaries and graphical representations of the risk of bias associated with the six RCTs are depicted in Fig. 2 as well as Fig. 3, respectively.

### Diagnostic accuracy

**Fig. 4:**
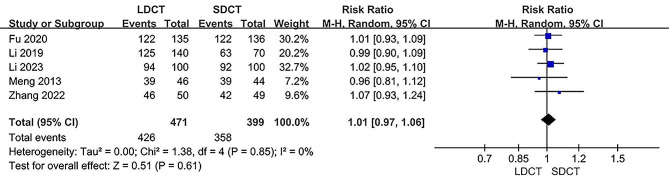
Forest plot for the meta-analysis of diagnostic accuracy in the ITT populations

**Fig. 5:**
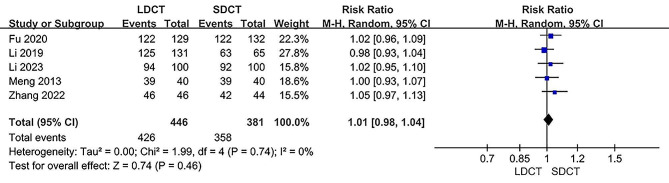
Forest plot for the meta-analysis of diagnostic accuracy in the PP populations

Data from five independent studies were utilized to assess diagnostic accuracy [12, 14–17]. There was no significant difference in diagnostic accuracy rates between the LDCT and SDCT groups within both the ITT populations (RR: 1.01, 95% CI 0.97–1.06, *P* = 0.61) and the PP populations (RR: 1.01, 95% CI: 0.98–1.04, *P* = 0.46). Heterogeneity was found to be non-significant (I^2^ = 0%, *P* = 0.85 and I^2^ = 0%, *P* = 0.74 respectively). Forest plots illustrating the meta-analysis of diagnostic accuracy in the ITT populations and PP populations are presented in Figs. 4 and 5, respectively.

### Radiation dose

**Fig. 6:**
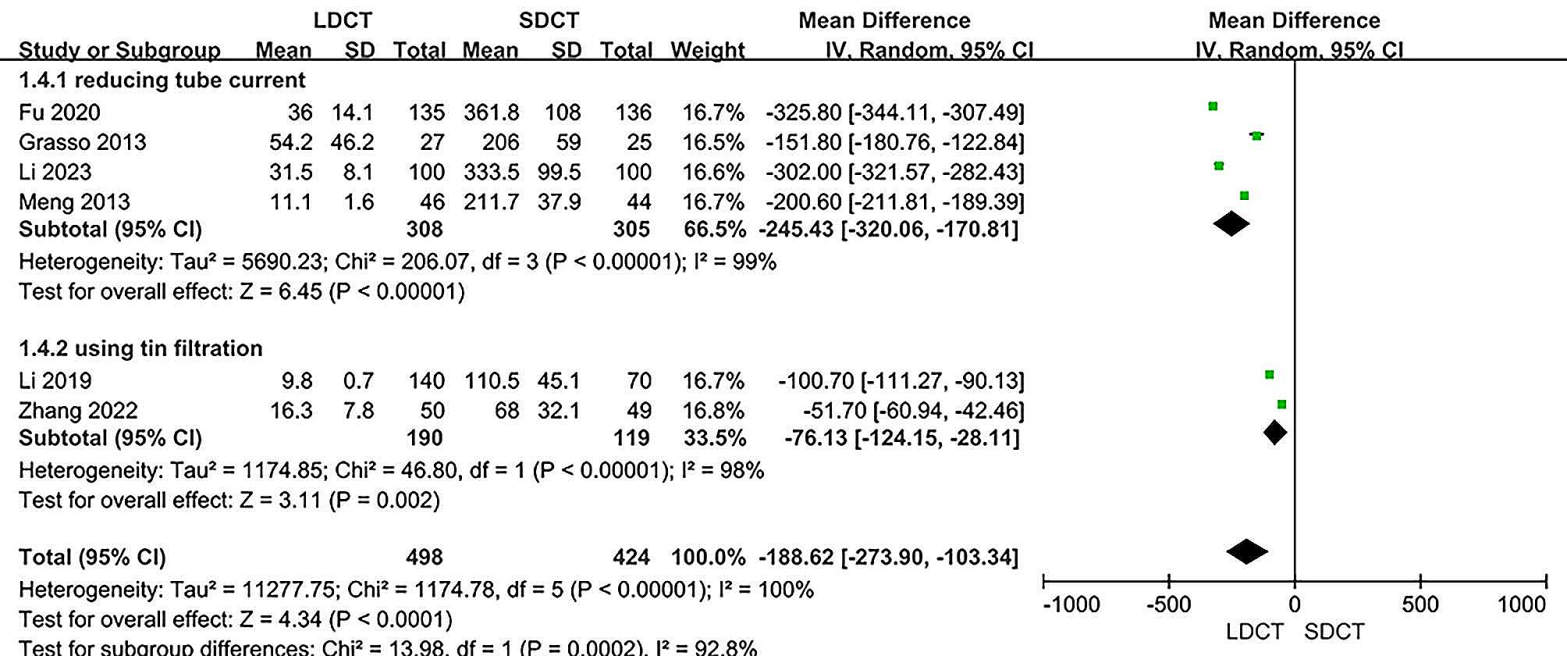
Forest plot for the meta-analysis of radiation dose

All selected studies contributed data related to DLP radiation dose assessment [11, 12, 14–17]. Compared with the SDCT group, DLP was notably lower for patients among the LDCT group (MD: -188.62, 95% CI: -273.90 to -103.34, *P* < 0.0001). There was significant heterogeneity existed among the included studies (I^2^ = 100%, *P* < 0.00001). An attempt was made to address this heterogeneity through subgroup analysis. It was postulated that the observed heterogeneity might have arisen from different approaches used to implement a low-dose protocol, such as reduced tube current or the use of tin filtration. However, significant heterogeneity persisted in both subgroups. Sensitivity analyses indicated that excluding individual studies did not alter the observed heterogeneity. Figure 6 illustrates the forest plot derived from the meta-analysis of the radiation dose (in mGy·cm).

### Operation duration

**Fig. 7:**
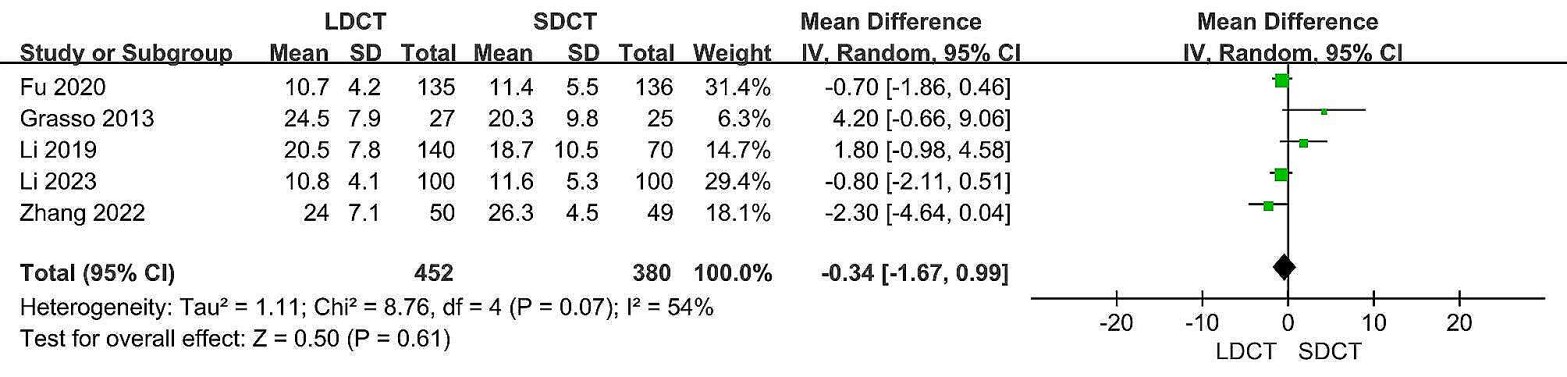
Forest plot for the meta-analysis of operation duration

Data regarding the duration of operations was obtained from five studies [11, 14–17], the results indicated that the operative time was not statistically different between the LDCT group and the SDCT group (MD: -0.34, 95% CI: -1.67 to 0.99, *p* = 0.61). However, we observed substantial heterogeneity (I^2^ = 54%, *p* = 0.07). To address this, sensitivity analyses were conducted, and upon excluding the Grass 2013 study, the heterogeneity reduced to 39%, resulting in a more consistent outcome (MD: -0.68, 95% CI: -1.78 to 0.41, *p* = 0.22) (Figure S1). Figure 7 displays the forest plot derived from the meta-analysis of operation duration (in minutes).

### Pneumothorax

**Fig. 8:**
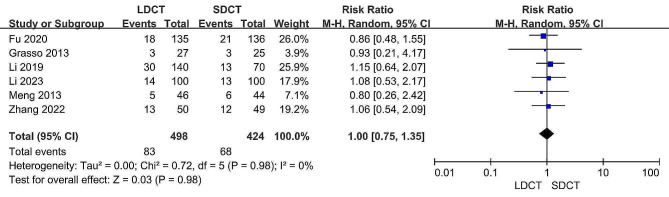
Forest plot for the meta-analysis of pneumothorax

All studies contributed data on pneumothorax rates [11, 12, 14–17]. Upon comparing the LDCT group with the SDCT group, no statistically significant differences were found in the pneumothorax rates (RR: 1.00, 95% CI: 0.75–1.35, *p* = 0.98). Additionally, there was no significant observed heterogeneity (I^2^ = 0%, *p* = 0.98). Figure 8 displays the forest plot generated from the meta-analysis of pneumothorax.

### Hemoptysis

**Fig. 9:**
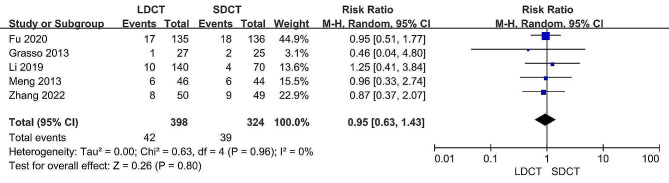
Forest plot for the meta-analysis of hemoptysis

Information regarding hemoptysis rates was retrieved from five distinct studies [11, 12, 14–16]. Subsequent analysis revealed that the hemoptysis rates were not statistically different between the LDCT and SDCT groups (RR: 0.95, 95% CI:0.63–1.43, *p* = 0.80). Furthermore, there was no significant heterogeneity existed (I^2^ = 0%, *p* = 0.96). Figure 9 illustrates the forest plot resulting from the meta-analysis of hemoptysis.

## Discussion

This study involved a meta-analysis comprising a total of 922 subjects collected from six independent articles. The purpose was to evaluate the effectiveness and safety of LDCT-guided lung biopsy, with a primary focus on assessing the diagnostic accuracy of the biopsy results. To further investigate the differences in diagnostic accuracy between the LDCT and SDCT groups, separate analyses were carried out on the ITT and PP populations. The findings indicated that the LDCT protocols demonstrated comparable diagnostic accuracy to the SDCT protocols for both ITT and PP populations. The combined diagnostic accuracy of LDCT-guided lung biopsy was determined to be 90.4% in the ITT analysis and 95.5% in the PP analysis, consistent with rates previously reported in CT-guided lung biopsy studies [1, 23].

In the present study, the impact on radiation dose—an additional endpoint—was examined, reflecting the widely accepted principle of minimizing unnecessary exposure in line with the As Low As Reasonably Achievable (ALARA) principle [24]. Various strategies currently exist to reduce CT radiation doses, including measures such as lowering tube voltage, reducing tube current, shortening scanning time, and employing iterative algorithms [25]. Another method to decrease radiation dose involves using tin filtration, which filters out many of the less dose-efficient low-energy photons that contribute significantly to patient exposure, thus reducing the delivered dose to the patient [26]. Within the scope of this meta-analysis, four studies opted to decrease the tube current, while the remaining two studies utilized tin filtration. It was observed that LDCT protocols were linked to lower doses of radiation. However, a notable heterogeneity existed among the included studies pertinent to this endpoint. In the present study, we could not identify sources of heterogeneity in either subgroup analysis or sensitivity analyses, potentially attributed to differences in types and models of CT scanners or scanning protocols employed by the included studies.

Significant statistical heterogeneity was noted in the duration of operations, with the exclusion of the Grass 2013 study resulting in a notable reduction in heterogeneity without altering the direction of the new pooled effect size. The Grass study utilized an optical navigation system, distinguishing it from the other studies, which may have contributed to the observed heterogeneity. The authors did not provide a comprehensive description of the biopsy procedure in the publication, precluding an assessment of potential additional factors contributing to clinical heterogeneity. The studies conducted by Fu 2020 and Li 2023 demonstrated a significant decrease in operation duration, potentially attributed to the higher levels of operator experience (15 years and 10 years, respectively) in comparison to the other studies (3 to 5 years). This discrepancy in operator experience may account for the observed variations in operation duration.

This study assessed the incidence of pneumothorax and hemoptysis as indicators of biopsy-related complications across two distinct groups. Equivalent rates of these complications were observed in the LDCT group as well as SDCT group. These results suggest that reducing the effective dose of radiation does not compromise the safety of the procedures of CT-guided lung biopsy.

Although the studies included suggested that the LDCT protocols yield inferior image quality [12, 14–16], a meta-analysis for this variable could not be performed due to inconsistencies in the strategies used for assessing image quality across the included studies. It appears that the relatively reduced image quality remains acceptable for the biopsy procedure. This can be attributed to several factors: [1] diagnostic CT images were obtained prior to the biopsy; [2] images from CT-guided procedures do not require spatial or contrast resolution equivalent to diagnostic images; [3] there exists favorable natural contrast along with low X-ray absorption rates associated with the lung.

With the advancement of image reconstruction techniques, LDCT protocols are expected to be more frequently utilized for guiding lung biopsies. Traditionally, the predominant reconstruction method has been the Filtered Back-Projection, which is noteworthy as a dose-dependent algorithm, necessitating a sufficiently high radiation dose to ensure an adequate signal-to-noise ratio [27]. However, in recent years, due to improved computer performance, the use of Iterative Reconstruction algorithms, previously limited by computational capabilities, has been growing [28]. Despite differences in the approaches toward these algorithms, their common feature lies in the ability to utilize data more efficiently, reduce noise and artifacts, and generate superior images from lower quality data [29, 30]. Furthermore, the emergence of deep learning-based reconstruction methods represents a significant leap forward, demonstrating considerable potential in enhancing image quality while minimizing radiation exposure [31].

The strength of our meta-analysis lies in the robustness and rigor of our methodology. We strictly adhered to the PRISMA guidelines and engaged two independent authors who worked in a double-blind manner. This approach enhanced the comprehensiveness of our systematic review, reducing the risk of overlooking relevant publications. To minimize potential data errors, two independent authors conducted quality assessments of each eligible trial and performed data extraction in a double-blind manner. Our meta-analysis included only randomized controlled studies that strictly adhered to precise inclusion and exclusion criteria, further bolstering the reliability and validity of our findings. Moreover, by assigning Yun Liu, an author lacking specific expertise in lung biopsy, to conduct the review, we reduced potential bias in evaluating study quality. This lack of prior exposure to these studies and independence from their results ensures a more impartial assessment.

Despite the meticulous nature of our methodology, our study faced several limitations. Firstly, the majority of the RCTs exhibited suboptimal methodological quality. Furthermore, a noticeable heterogeneity was evident in the analysis of radiation dose. Despite conducting subgroup and sensitivity analyses, discerning the root cause of this heterogeneity remained elusive. Moreover, our study rested upon merely six RCTs, raising concerns about potential bias in the pooled effect due to unpublished or missing data, significantly challenging the generalizability of our findings. Consequently, further research involving well-designed and larger population is imperative to firmly establish the safety as well as the diagnostic efficacy of LDCT-guided lung biopsies compared to SDCT-guided lung biopsies.

## Conclusion

LDCT-guided lung biopsies demonstrated comparable outcomes to SDCT-guided lung biopsies concerning diagnostic accuracy, operation duration, and complication incidence. Notably, LDCT-guided lung biopsies offered a substantial reduction in radiation exposure, underscoring their potential advantages.

### Electronic supplementary material

Below is the link to the electronic supplementary material.

Supplementary Material 1

Supplementary Material 2

## Data Availability

The datasets used or analyzed during the current study are available from the corresponding author on reasonable request.

## Abbreviations

RCTs: Randomized Controlled Trials
CT: Computed tomography
LDCT: Low-dose computed tomography
SDCT: Standard-dose computed tomography
BMI: Body mass index
ITT: Intent-to-treat; PP: Per-protocol
RR: Relative risk
CI: Confidence intervals
MD: Mean differences

## References

[1] Li Y, Yang F, Huang YY, Cao W (2022). Comparison between computed tomography-guided core and fine needle lung biopsy: a meta-analysis. Medicine.

[2] Wallace MJ, Krishnamurthy S, Broemeling LD, Gupta S, Ahrar K, Morello FA (2002). CT-guided percutaneous fine-needle aspiration biopsy of small (< or = 1-cm) pulmonary lesions. Radiology.

[3] Tian P, Wang Y, Li L, Zhou Y, Luo W, Li W (2017). CT-guided transthoracic core needle biopsy for small pulmonary lesions: diagnostic performance and adequacy for molecular testing. J Thorac Disease.

[4] Maybody M, Muallem N, Brown KT, Moskowitz CS, Hsu M, Zenobi CL (2019). Autologous blood Patch Injection versus Hydrogel Plug in CT-guided lung biopsy: a prospective Randomized Trial. Radiology.

[5] Heerink WJ, de Bock GH, de Jonge GJ, Groen HJ, Vliegenthart R, Oudkerk M (2017). Complication rates of CT-guided transthoracic lung biopsy: meta-analysis. Eur Radiol.

[6] Marshall D, Laberge JM, Firetag B, Miller T, Kerlan RK (2013). The changing face of percutaneous image-guided biopsy: molecular profiling and genomic analysis in current practice. J Vascular Interventional Radiology: JVIR.

[7] Johnson JN, Hornik CP, Li JS, Benjamin DK, Yoshizumi TT, Reiman RE (2014). Cumulative radiation exposure and cancer risk estimation in children with heart disease. Circulation.

[8] Fazel R, Krumholz HM, Wang Y, Ross JS, Chen J, Ting HH (2009). Exposure to low-dose ionizing radiation from medical imaging procedures. N Engl J Med.

[9] Liu QX, Zhou D, Han TC, Lu X, Hou B, Li MY (2021). A Noninvasive Multianalytical Approach for Lung Cancer diagnosis of patients with pulmonary nodules. Adv Sci (Weinheim Baden-Wurttemberg Germany).

[10] Gao Y, Hua M, Lv J, Ma Y, Liu Y, Ren M (2022). Reproducibility of radiomic features of pulmonary nodules between low-dose CT and conventional-dose CT. Quant Imaging Med Surg.

[11] Grasso RF, Cazzato RL, Luppi G, D’Agostino F, Schena E, Del Vescovo R (2013). Percutaneous lung biopsies: performance of an optical CT-based navigation system with a low-dose protocol. Eur Radiol.

[12] Meng XX, Kuai XP, Dong WH, Jia NY, Liu SY, Xiao XS. Comparison of lung lesion biopsies between low-dose CT-guided and conventional CT-guided techniques. Acta radiologica (Stockholm, Sweden: 1987). 2013;54(8):909 – 15. 10.1177/0284185113485937 23817682

[13] Coppola G, Iezzi R, Posa A, Antonuccio EGM, Congedo MT, Bonomo L (2016). CT-guided needle biopsy of lung lesions: is there the possibility of reducing the dose?. Cardiovasc Interv Radiol.

[14] Li C, Liu B, Meng H, Lv W, Jia H (2019). Efficacy and Radiation exposure of ultra-low-dose chest CT at 100 kVp with tin filtration in CT-Guided percutaneous core needle biopsy for small pulmonary lesions using a third-generation dual-source CT scanner. J Vascular Interventional Radiology: JVIR.

[15] Fu YF, Li GC, Xu QS, Shi YB, Wang C, Wang T (2020). Computed tomography-guided lung biopsy: a randomized controlled trial of low-dose versus standard-dose protocol. Eur Radiol.

[16] Zhang J, Liu ML, Liu DH, Li XQ, Lin M, Tan Y (2022). Low-dose CT with tin filter combined with iterative metal artefact reduction for guiding lung biopsy. Quant Imaging Med Surg.

[17] Li EL, Ma AL, Wang T, Fu YF, Liu HY, Li GC (2023). Low-dose versus standard-dose computed tomography-guided biopsy for pulmonary nodules: a randomized controlled trial. J Cardiothorac Surg.

[18] Zhang P, Liu JM, Zhang YY, Hua R, Xia FF, Shi YB (2021). Computed tomography-guided lung biopsy: a meta-analysis of low-dose and standard-dose protocols. J Cancer Res Ther.

[19] Moher D, Liberati A, Tetzlaff J, Altman DG (2009). Preferred reporting items for systematic reviews and meta-analyses: the PRISMA statement. BMJ.

[20] Higgins JP, Altman DG, Gøtzsche PC, Jüni P, Moher D, Oxman AD (2011). The Cochrane collaboration’s tool for assessing risk of bias in randomised trials. BMJ.

[21] Higgins JP, Thompson SG (2002). Quantifying heterogeneity in a meta-analysis. Stat Med.

[22] Sterne JA, Sutton AJ, Ioannidis JP, Terrin N, Jones DR, Lau J (2011). Recommendations for examining and interpreting funnel plot asymmetry in meta-analyses of randomised controlled trials. BMJ (Clinical Res ed).

[23] Zhao G, Shi X, Sun W, Liang H, Mao X, Wen F (2017). Factors affecting the accuracy and safety of computed tomography-guided biopsy of intrapulmonary solitary nodules ≤ 30 mm in a retrospective study of 155 patients. Experimental Therapeutic Med.

[24] Xie Z, Liao X, Kang Y, Zhang J, Jia L (2016). Radiation exposure to staff in Intensive Care Unit with portable CT scanner. Biomed Res Int.

[25] Kalra MK, Maher MM, Toth TL, Hamberg LM, Blake MA, Shepard JA (2004). Strategies for CT radiation dose optimization. Radiology.

[26] Weis M, Henzler T, Nance JW, Haubenreisser H, Meyer M, Sudarski S (2017). Radiation Dose Comparison between 70 kVp and 100 kVp with Spectral Beam shaping for Non-contrast-enhanced Pediatric chest computed tomography: a prospective randomized controlled study. Invest Radiol.

[27] Schofield R, King L, Tayal U, Castellano I, Stirrup J, Pontana F (2020). Image reconstruction: part 1 - understanding filtered back projection, noise and image acquisition. J Cardiovasc Comput Tomogr.

[28] Staniszewska M, Chrusciak D (2017). Iterative Reconstruction as a method for optimisation of computed tomography procedures. Pol J Radiol.

[29] Geyer LL, Schoepf UJ, Meinel FG, Nance JW, Bastarrika G, Leipsic JA (2015). State of the art: Iterative CT Reconstruction techniques. Radiology.

[30] Lauzier PT, Chen GH (2013). Characterization of statistical prior image constrained compressed sensing (PICCS): II. Application to dose reduction. Med Phys.

[31] Li M, Nyayapathi N, Kilian HI, Xia J, Lovell JF, Yao J (2020). Sound out the deep colors: Photoacoustic Molecular Imaging at New depths. Mol Imaging.

