# Supplementary figures and images for: A systematic review and meta-analysis of randomized controlled trials comparing low-dose versus standard-dose computed tomography-guided lung biopsy
Source: J Cardiothorac Surg. 2024 May 22;19:297. doi: 10.1186/s13019-024-02792-x (PMC11110412; doi:10.1186/s13019-024-02792-x)

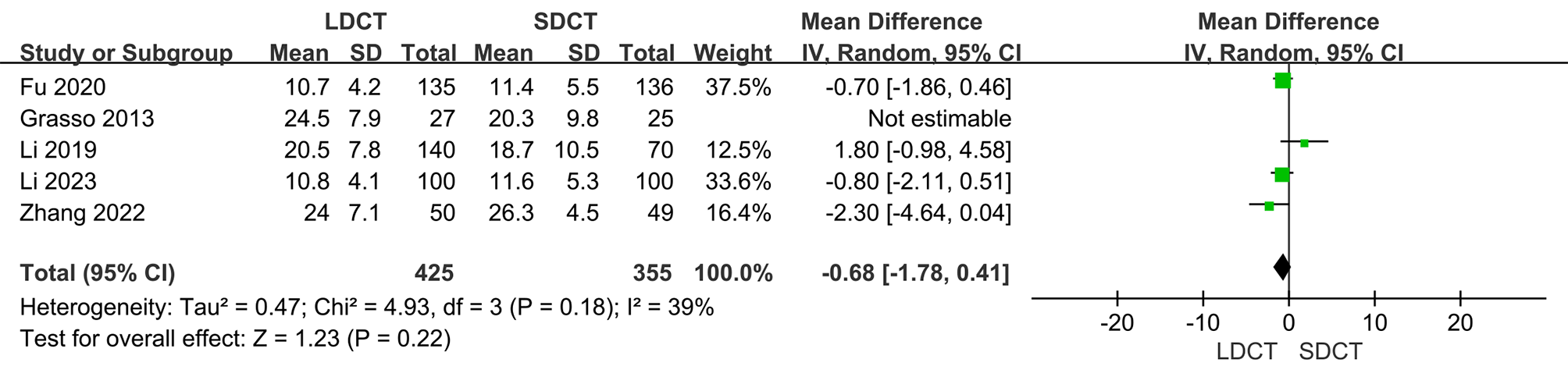

Supplement: Supplementary file 1 — Supplementary Material 1 [file 13019_2024_2792_MOESM1_ESM.tif]
