# Supplementary material for: A systematic review and meta-analysis of randomized controlled trials comparing low-dose versus standard-dose computed tomography-guided lung biopsy
Source: J Cardiothorac Surg. 2024 May 22;19:297. doi: 10.1186/s13019-024-02792-x (PMC11110412; doi:10.1186/s13019-024-02792-x)
Supplement: Supplementary file 2 — Supplementary Material 2 [file 13019_2024_2792_MOESM2_ESM.docx]

**Search strategy for PubMed**

1. "tomography, x ray computed"[MeSH Terms] 492,064
2. "CT"[Title/Abstract] OR "computed tomography"[Title/Abstract] OR "computerized tomography"[Title/Abstract] OR "tomography x ray computed"[Title/Abstract] 627,985
3. "tomography, x ray computed"[MeSH Terms] OR "CT"[Title/Abstract] OR "computed tomography"[Title/Abstract] OR "computerized tomography"[Title/Abstract] OR "tomography x ray computed"[Title/Abstract] 830,662
4. "low-dose"[Title/Abstract]" OR "low dose"[Title/Abstract] 114986
5. (("Tomography, X-Ray Computed"[Mesh]) OR (CT [Title/Abstract] OR Computed Tomography [Title/Abstract] OR Computerized Tomography [Title/Abstract] OR Tomography, X-Ray Computed [Title/Abstract])) AND ((low dose[Title/Abstract]) OR (low-dose[Title/Abstract])) 10233
6. "LDCT"[Title/Abstract] 1531
7. ((("Tomography, X-Ray Computed"[Mesh]) OR (CT [Title/Abstract] OR Computed Tomography [Title/Abstract] OR Computerized Tomography [Title/Abstract] OR Tomography, X-Ray Computed [Title/Abstract])) AND ((low dose[Title/Abstract]) OR (low-dose[Title/Abstract]))) OR (LDCT[Title/Abstract]) 10329
8. "Pulmonary"[Title/Abstract] OR "lung"[Title/Abstract] 1187146
9. "biops*"[Title/Abstract] 467606
10. ("Pulmonary"[Title/Abstract] OR "lung"[Title/Abstract]) AND "biops*"[Title/Abstract] 42487
11. (((("Tomography, X-Ray Computed"[Mesh]) OR (CT [Title/Abstract] OR Computed Tomography [Title/Abstract] OR Computerized Tomography [Title/Abstract] OR Tomography, X-Ray Computed [Title/Abstract])) AND ((low dose[Title/Abstract]) OR (low-dose[Title/Abstract]))) OR (LDCT[Title/Abstract])) AND (((Pulmonary[Title/Abstract]) OR (lung[Title/Abstract])) AND (biops*[Title/Abstract])) 285
12. Controlled clinical trial[Publication Type] OR randomized[Title/Abstract] OR randomly[Title/Abstract] OR trial[Title/Abstract] OR groups[Title/Abstract] OR random[Title/Abstract] OR "Randomized Controlled Trial" [Publication Type] 4038246
13. ((randomized controlled trial[pt] OR controlled clinical trial[pt] OR randomized[tiab] OR placebo[tiab] OR non drug therapy[sh] OR randomly[tiab] OR trial[tiab] OR groups[tiab]) NOT (animals[mh] NOT humans[mh])) AND (((randomized controlled trial[pt] OR controlled clinical trial[pt] OR randomized[tiab] OR placebo[tiab] OR non drug therapy[sh] OR randomly[tiab] OR trial[tiab] OR groups[tiab]) NOT (animals[mh] NOT humans[mh])) AND ((((("Tomography, X-Ray Computed"[Mesh]) OR (CT [Title/Abstract] OR Computed Tomography [Title/Abstract] OR Computerized Tomography [Title/Abstract] OR Tomography, X-Ray Computed [Title/Abstract])) AND ((low dose[Title/Abstract]) OR (low-dose[Title/Abstract]))) OR (LDCT[Title/Abstract])) AND (((Pulmonary[Title/Abstract]) OR (lung[Title/Abstract])) AND (biops*[Title/Abstract])))) 94

**Search strategy for EMbase**

1. 'x-ray computed tomography'/exp OR 'x-ray computed tomography' 106,272
2. 'ct':ab,ti OR 'computed tomography':ab,ti OR 'computerized tomography':ab,ti 956,720
3. 1 OR 2 991,533
4. 'low dose':ab,ti OR 'low-dose':ab,ti 167,085
5. 3 AND 4 17,255
6. 'low-dose computed tomography'/exp OR 'low-dose computed tomography' 4,149
7. 'ldct':ab,ti 2,736
8. 5 OR 6 OR 7 18,025
9. 'pulmonary':ab,ti OR 'lung':ab,ti 1,678,863
10. biops*:ab,ti 760,742
11. 9 AND 10 83,295
12. 'randomized controlled trial' OR random*:ab,ti 2,176,561
13. 8 AND 11 AND 12 73

**Search strategy for Cochrane Library**

1. MeSH descriptor: [Tomography, X-Ray Computed] explode all trees 7267
2. (ct):ti,ab,kw OR (computed tomography):ti,ab,kw OR (computerized tomography):ti,ab,kw OR (Tomography, X-Ray Computed):ti,ab,kw 94638
3. 1 OR 2 94678
4. (low dose):ti,ab,kw OR (low-dose):ti,ab,kw 67865
5. 3 AND 4 8100
6. LDCT 266
7. 5 OR 6 8131
8. (Pulmonary):ti,ab,kw OR (lung):ti,ab,kw 116874
9. (biops*):ti,ab,kw 37010
10. 8 AND 9 2756
11. 7 AND 10 77

**Search strategy for Web of Science**

1: TS=(x-ray computed tomography OR ct OR computed tomography OR computerized tomography) 711187

2: TS=(low dose OR low-dose ) 465238

3: 2 AND 1 23515

4: TS=(low-dose computed tomography OR low dose computed tomography OR LDCT ) 8461

5: 3 OR 4 23685

6: TS=(pulmonary OR lung ) 1328570

7: TS=(biops* ) 423529

8: 6 AND 7 40457

9: TS=(randomized controlled trial OR random* OR RCT) 2139801

10: 9 AND 8 AND 5 44

**Search strategy for ClinicalTrials.gov**

Condition/disease :lung biopsy or pulmonary biopsy

Intervention/treatment: low dose CT or LDCT 4
